# Molecular Detection of *Leishmania* spp. and Blood Source of Female Sand Flies in the Parque Estadual do Rio Doce and Municipality of Timóteo, Minas Gerais, Brazil

**DOI:** 10.3390/tropicalmed9060133

**Published:** 2024-06-13

**Authors:** Cristian Ferreira de Souza, Carlos Alberto dos Santos, Paula Dias Bevilacqua, José Dilermando Andrade Filho, Reginaldo Peçanha Brazil

**Affiliations:** 1 Laboratório de Doenças Parasitárias, Instituto Oswaldo Cruz (Fiocruz), Avenida Brasil, 4365, Manguinhos, Rio de Janeiro 21040-360, RJ, Brazil; biominas2004@yahoo.com.br; 2 Centro de Controle de Zoonoses, Prefeitura Municipal de Timóteo, Avenida Acesita, 3230, São José, Timóteo 35182-000, MG, Brazil; carlos_alberto.tec@hotmail.com; 3 Grupo de Pesquisa Violências, Gênero e Saúde, Instituto René Rachou (Fiocruz), Avenida Augusto de Lima, 1715, Barro Preto, Belo Horizonte 30190-002, MG, Brazil; paula.bevilacqua@fiocruz.br; 4 Grupo de Estudos em Leishmanioses, Instituto René Rachou (Fiocruz), Avenida Augusto de Lima, 1715, Barro Preto, Belo Horizonte 30190-002, MG, Brazil; jose.andrade@fiocruz.br

**Keywords:** leishmaniasis, vectors, blood source, sand flies, *Leishmania*

## Abstract

Leishmaniasis is a group of diseases caused by protozoa of the genus *Leishmania* and is transmitted by the bite female sand fly. The present work is characterized as a descriptive study in two areas: a forest area located in the Parque Estadual do Rio Doce, and another urban area located in the municipality of Timóteo-MG, with the objective of identifying the presence of *Leishmania* spp. and the blood source of the collected female sand flies. Part of the females were obtained from the Parque Estadual do Rio Doce, and part was collected using 19 ligth traps distributed in residences of Timóteo. For molecular studies of *Leishmania* spp. DNA, the ITS1 gene was used, and in the search for blood source, the CytB gene was used and positive samples were sequenced. The study demonstrated that there are at least three species of *Leishmania* circulating in the study areas: *Leishmania* (*Viannia*) *braziliensis*, *Leishmania* (*Leishmania*) *amazonensis*, and *Leishmania* (*V*.) *guyanensis*. *Nyssomyia whitmani* was the predominant sand fly species in the urban area of Timóteo with a positive diagnosis for the presence of *Leishmania braziliensis* DNA. We found the presence of blood from *Gallus gallus* (Chicken) and *Sus scrofa* (Pig) in sand flies. The present study demonstrates that *Leishmania braziliensis* is the main agent of cutaneous leishmaniasis in the study area, with the effective participation of *Nyssomyia whitmani* as the vector and both *Gallus gallus* and *Sus scrofa* acting as a food source for female sand flies, and helping maintaining the sand fly life.

## 1. Introduction

Leishmaniases are enzootic and zoonotic diseases caused by morphologically similar parasitic protozoa of the genus *Leishmania* (Kinetoplastida: Trypanosomatidae) and transmitted by the bite of female insects called sand flies (Diptera: Psychodidae) [1]. In 2018, 92 and 83 countries were considered endemic or had already reported cases of tegumentary leishmaniasis (TL) and visceral leishmaniasis (VL), respectively. It is estimated that more than 1 billion people live in endemic areas for leishmaniasis and are at risk of infection, and that 30,000 new cases of VL and more than 1 million new cases of TL occur annually [2].

Data from World Health Organization [2] showed that in 2019, Brazil was one of the countries with the highest number of cases of VL and TL worldwide. Between 2010 and 2019, Brazil presented an annual average of 20,106 cases of TL and 36,432 cases of VL notified. In the state of Minas Gerais, with a population of 20,539,989 people [3], in the same period, 4919 cases of VL and 15,134 cases of TL were registered, with an average annual incidence of 7.4/100,000 inhabitants for TL and 2.4/100,000 inhabitants for VL [4]. In the municipality of Timóteo, 229 cases of TL were registered in the period from 2001 to 2019, and the first reported case of VL was in 2006, reaching a total of 21 cases in 2019 [4].

Regarding this municipality, species indicated as vectors of *Leishmania* have already been recorded, both in forested areas (Parque Estadual do Rio Doce—PERD) [5] and urban environments [6,7]. The main potential vector species recorded were *Lutzomyia longipalpis*, *Nyssomyia intermedia*, *Nyssomyia whitmani*, and *Migonemyia migonei* [8,9,10,11,12]. Although possible vector species have been identified, nothing is known about the *Leishmania* species that may be circulating in the municipality of Timóteo, not even the possible sources of blood for female sand flies.

In this sense, we determined, through molecular techniques, the blood source of naturally fed female sand flies, as well as the parasite species that can act as leishmaniasis agents in the study areas, thus providing support for a more effective prevention and control actions by health agencies.

## 2. Materials and Methods

### 2.1. Characterization of the Study Area

The study was carried out in the urban area of the municipality of Timóteo (19°34′58″ S; 42°38′38″ W) and in a forested area, Parque Estadual do Rio Doce (PERD). The municipality of Timóteo is located in the Metropolitan Region of Vale do Aço, in the state of Minas Gerais, with a land area of 145,159 km^2^. The PERD (19°42′23″ S; 42°34′33″ W) was created by Decree-Law nº 1.119, on 14 July 1944, being the first official conservation unit in the state of Minas Gerais, inserted between the municipalities of Timóteo, Marliéria, and Dionísio. It is one of the largest Atlantic Forest conservation areas in Brazil, with 35,976 hectares [3].

### 2.2. Sand Flies Used

**Figure 1:**
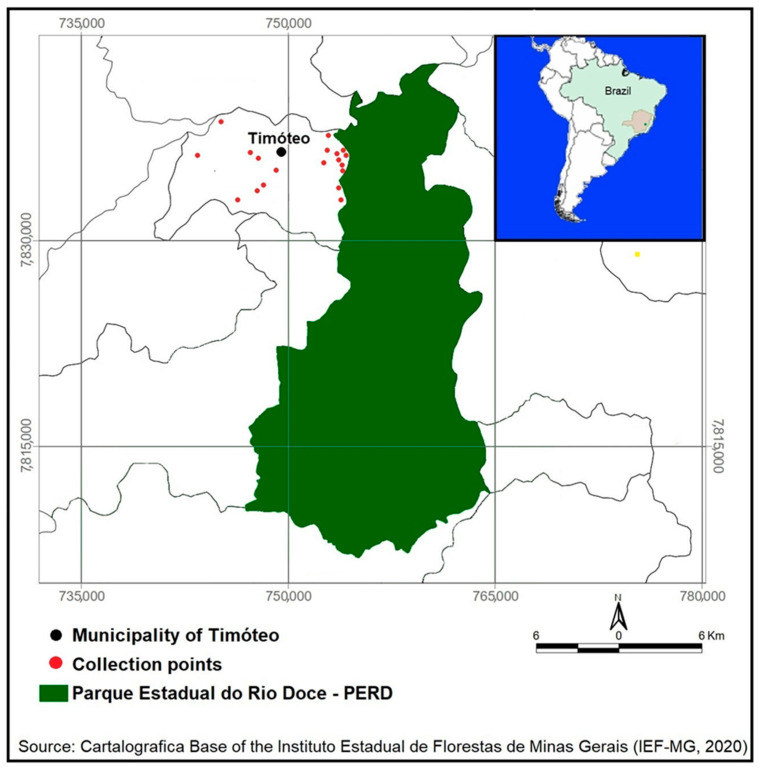
Map of the municipality of Timóteo showing the 19 collection points (●) of sand flies located in the urban area of the municipality; and (■) is an illustration of Parque Estadual do Rio Doce, where part of the female sand flies used in the study were collected. The two areas are located in the state of Minas Gerais, Brazil.

In the present study, female sand flies collected in PERD and in Timóteo were used for detection of *Leishmania* spp. DNA and for food-source study. The females used in the study and collected at PERD were previously described by Souza and Collaborators [5]. Already the sand flies of the urban area of Timóteo were captured in the peridomicile of 19 residences, with previous notification of human cases of leishmaniasis (data obtained from the Timóteo health department) and reported in previous studies with the presence of sand flies [7]. Collections were carried out with light traps, model HP, and were used between September 2012–February 2014. The traps were exposed for two consecutive nights (06:00 p.m. to 06:00 a.m.) with monthly collections, both in the peridomicile environment in Timóteo (Figure 1). 

The captured sand flies were killed in a refrigerated chamber for material screening. All males and part of the females were clarified, mounted in balsam medium between slides and cover slips, and identified. And another part of the females was dissected, and the head and last segments of the abdomen were used for identification. The rest of the female body (abdomen, thorax) was used for molecular studies. Females without any traces of blood in the abdomen were used to detect the presence of *Leishmania* spp. DNA. Females with traces of blood were used to verify the food source. The identification of species collected followed the classification proposed by Galati [13], and the abbreviations of the species names were made following a proposal by Marcondes [14].

### 2.3. Detection of Leishmania spp. DNA

Female sand flies were individually identified and stored in 1.5 mL polypropylene microtubes with 6% DMSO and used for DNA extraction using a commercial Gentra Puregene^®^ Cell and Tissue Extraction Kit (QIAGEN, Hilden, Germany) following the manufacturer’s protocol.

The extracted DNA was resuspended in 20 μL in rehydration solution (obtained from the extraction kit) and the microtubes were preserved at −20 °C. The total DNA samples extracted from female sand flies reached an average final concentration of 6 ng/μL.

The extracted DNA was submitted to the PCR technique for amplification of a target region of the DNA of *Leishmania* spp., the ITS1 (internal transcribed spacer 1) (LITSR 5′ CTGGATCATTTTCCGATG 3′ and L5.8S 5′ TGATACCACTTATCGCACTT 3′) [15].

In the PCR, the amplification of a fragment of approximately 350 bp was obtained using the following reaction: 1× buffer solution (200 mM Tris-HCl pH8.4, 500 mM KCl), 1.5 mM MgCl_2_, 0.2 mM dNTPs mixture, 0.5 pmol of LITSR primer, 0.5 pmol of L5.8S primer, 1 U of Taq DNA Polymerase Platinum^®^ (Invitrogen, Carlsbad, CA, USA), and 5 µL of template DNA, in a final volume of 25 µL. Amplification was performed alternating 33 cycles of denaturation at 95 °C for 30 s, annealing at 53 °C for 1 min, and extension at 72 °C for 1 min in an automatic DNA thermocycler equipment (MaxyGene Gradient—AXYGENE^®^, Corning, NY, USA). The amplified band profiles were analyzed on a 2% agarose gel stained with GelRed™ (Nucleic Acid Gel Stain—Biotium, Fremont, CA, USA), concentration used: GelRed 10,000× in 500 µL of contamination-free water (1:500). Then, we applied 1 µL of diluted GelRed™, 1 µL of loading buffer and 5 µL of amplified PCR product, and compared to PCR product from reference strains of *Leishmania braziliensis* (MHOM/BR/75/M2903). DNA from male sand flies were used as negative controls.

The PCR/ITS1 positive samples were cloned by bacterial transformation, using the *Escherichia coli* species (DH5-α) as competent bacteria cells, and for the ligation reaction the commercial CloneJet PCR Kit (Thermo Fisher Scientific, Carlsbad, CA, USA) was used; the preparation of the reaction followed the manufacturer’s protocol. Cloning was necessary to increase the amount of DNA amplified in PCR/ITS1 and subsequent sequencing of the samples.

### 2.4. Study of Blood Source

The DNA of female sand flies with signs of blood meal in their abdomen were analyzed using PCR and sequenced. The Cytochrome B (CytB) gene was used in the PCR (FOR5′-CCATCCAACATCTCAGCATGATGAAA-3′ and REV5′-GCCCCTCAGAATGATATTTGTCCTCA-3′) [16]. The DNA was subjected to PCR in a 1 × buffer solution (200 mM Tris-HCl pH8.4, 500 mM KCl), 1.5 mM MgCl_2_, 0.2 mM dNTPs mix, 0.5 pmol of CytB FOR5 primer and REV5, 1 U of platinum^®^ Taq DNA polymerase (Invitrogen), and 5 µL of template DNA in a final volume of 50 µL. Amplification was performed alternating 32 cycles of denaturation at 95 °C for 20 s, annealing at 53 °C for 30 s, and extension at 72 °C for 1 min in an automatic DNA thermocycler equipment (MaxyGene Gradient—AXYGENE^®^). The amplified band profile was analyzed on a 2% agarose gel stained with GelRed™, concentration used: GelRed 10,000 × in 500 µL of contamination-free water (1:500). Then, 1 µL of diluted GelRed™ was applied, in addition to 1 µL of loading buffer and 5 µL of amplified product in the PCR, and then was compared to amplified samples of DNA extraction from dog and rodent blood as a positive control. The material amplified in the CytB PCR was sequenced.

### 2.5. Sequencing

All samples that had amplified PCR product, both in the *Leishmania* spp. DNA detection study and in the blood source study, were sent for sequencing to the Macrogen^®^ company (Seoul, South Korea), where the automatic sequencer (Applied Biosystems 3730XL, Thermo Fisher Scientific, Carlsbad, CA, USA) was used. Each sample had its DNA sequenced in both directions. The sequences obtained were aligned and edited using the Sequencher^®^ 4.1.4 program, and compared using the Blast algorithm (Basic Local Alignment Search Tool) with sequences available in GenBank.

### 2.6. Data Analysis

The data presented were worked using the calculating proportions of species, sex, and presence or absence of *Leishmania* spp. DNA. The detection rate (DT) of *Leishmania* spp. DNA was calculated using the number of positive samples divided by the total number of samples and multiplied by 100. Analyses involving calculations of proportions were performed using the Microsoft^®^ Office Excel 2016 program, and after the normality test using the Shapiro–Wilk test. Comparisons between proportions were obtained using the Z test tabulated in Microsoft^®^ Office Excel 2016, and analyzed in STATA^®^12 and XLSTAT^®^15. The interpretations were made adopting a significance level of 5% (α = 0.05).

## 3. Results

### 3.1. Sand Flies Collected in Timóteo

A total of 3942 sandflies were collected in the urban area of Timóteo (Table 1), distributed in 22 species, with a greater number of females 2692 (68.29%) compared to males 1250 (31.71%) (z = 32.48 *p* < 0.0001). The most abundant species were *Nyssomyia whitmani* (66.49%) and *Nyssomyia intermedia* (18.21%), both species identified as vectors in TL (8, 11). *Lutzomyia longipalpis*, species identified as a vector for VL (10), was also collected, but in smaller numbers (0.18%).

### 3.2. Molecular Detection of Leishmania spp. DNA

**Figure 2:**
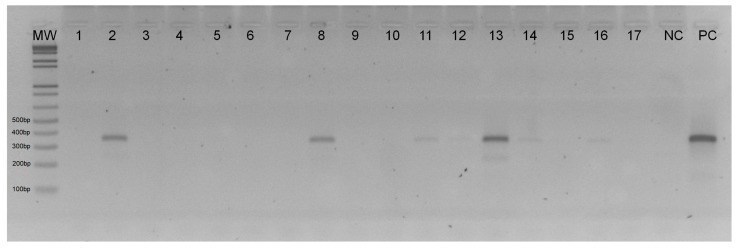
Detection of *Leishmania* spp. DNA using PCR/ITS1 in total DNA from female sand fly samples. The figure represents a 2% agarose gel stained with GelRed™ and subjected to electrophoresis. Lanes: MW—Molecular weight marker (100 bp); 2, 8, 11, 12, 13, 14, and 16—Positive samples for *Leishmania* spp. DNA; 1, 3, 4, 5, 6, 7, 9, 10, 15, and 17—Negative samples for *Leishmania* spp. DNA; e NC—Negative control; PC—Positive control of *Leishmania braziliensis* (MHOM/BR/75/M2903).

A total of 2939 female sand flies without the presence of blood in the abdomen were used in the detection of *Leishmania* spp. DNA. Of these, 370 (12.6%) were captured in the PERD and 2569 (87.4%) in Timóteo (Table 2). After PCR/ITS1, 57 samples were detected with *Leishmania* spp. DNA, 6 being collected in the PERD and 51 in Timóteo (Figure 2, Table 2).

The DNA detection rate was 1.62% for PERD and 1.94% for Timóteo. The species *Psychodopygus davisi* (1.30%) *Pressatia choti* (3.81%) were the only ones detected with *Leishmania* spp. DNA in the PERD. In relation to the Timóteo samples, among the species recognized as vectors, the highest DNA detection rate were *Migonemyia migonei* (3.30%), *Nyssomyia whitmani* (2.07%), and *Nyssomyia intermedia* (1.39%) (Table 2).

**Figure 3:**
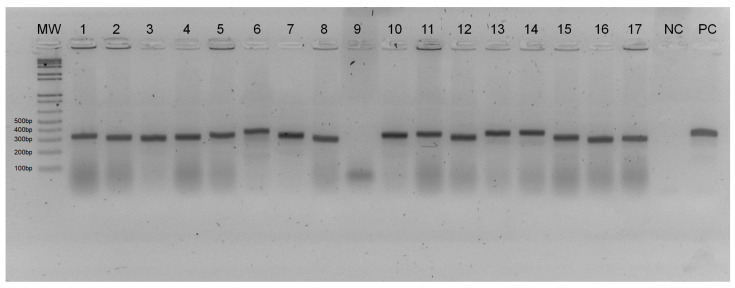
Detection of *Leishmania* spp. DNA in the cloned samples using PCR/ITS1. The figure represents a 2% agarose gel stained with GelRed™ and subjected to electrophoresis. Lanes: MW—Molecular weight marker (100 bp); 1, 2, 3, 4, 5, 6, 7, 8, 10, 11, 12, 13, 14, 15, 16, and 17—Clone of positive samples of *Leishmania* spp. DNA; 9—Non-cloned sample; e NC—Negative control; and PC—Positive control of *Leishmania braziliensis* (MHOM/BR/75/M2903).

A total of 49 PCR/ITS1 positive samples were cloned (Figure 3), sequenced, and deposited in GenBank under numbers MT707557–MT707605. Of the 49 samples sequenced, two (4.08%) female sand flies of the species *Pressatia choti* were collected at PERD with *Leishmania braziliensis* DNA detected. The other samples were collected in Timóteo. The species with the highest numbers of females detected with *Leishmania* spp. DNA was *Nyssomyia whitmani* with 35 (71.43%) samples identified with the presence of *Leishmania braziliensis* DNA, followed by *Nyssomyia intermedia* with 06 (12.24%) samples with the presence of *Leishmania braziliensis* DNA. Also in Timóteo, one specimen of *Migonemyia migonei* and one of *Nyssomyia intermedia* with the presence of *Leishmania amazonensis* DNA, and a specimen of *Nyssomyia intermedia* with the presence of *Leishmania guyanensis* DNA, were identified (Table 3).

### 3.3. Blood Source Study

PCR/CytB was performed on 44 fed females of sand flies with the presence of blood in the abdomen (Table 4), 39 (88.6%) were collected in Timóteo and 5 (11.4%) in the PERD, followed by sequencing of the samples and deposited in GenBank under numbers MT796689–MT796732. In the samples analyzed, blood DNA from six species was amplified: one bird species, *Gallus gallus* (Chicken), and other five species of mammals: *Dasyproca leporina* (Agouti), *Dasypus novemcinctus* (Armadillo), *Sphiggurus villosus* (Hedgehog), *Sus crofa* (Pig), and *Homo sapiens* (Table 4).

Among the specimens of female sand flies collected in the PERD, genetic material of *Dasyprocta leporina*, *Dasypus novemcinctus*, *Sphiggurus villosus*, and *Homo sapiens* was identified. Regarding the 39 female sand flies collected in Timóteo, a blood source was identified as *Homo sapiens* (*n* = 6/15.4%), *Gallus gallus* (*n* = 22/56.4%), and *Sus scrofa* (*n* = 11/28.2%).

## 4. Discussion

### 4.1. Sand Flies Collected in Timóteo

The large number of specimens collected in Timóteo, when compared to the number of sand flies collected by Souza and Collaborators in the PERD (1993 specimens of sand flies) (z = 35.78 *p* < 0.0001) [5], associated with the diversity of sand flies species (22 species), including vectors, may be related the ability of these sand flies to adapt to anthropogenic environments, and the availability of food and ideal shelter for the maintenance and survival of sand flies in Timóteo, corroborating reports from other studies [17].

Among the most collected species, *Nyssomyia intermedia* and *Migonemyia migonei* are already described as species with a high degree of anthropophily and high capacity to adapt to the man modified environment [18,19]. *Nyssomyia whitmani*, on the other hand, has been shown to be a species with greater tendency to the transitional environment, and can adapt to the peridomicile [20]. The large number of these species collected in Timóteo demonstrates that they are already adapted to the city’s urban environment. And the fact that these species are of medical importance requires special attention from health authorities. Although it is not the first record of *Lutzomyia longipalpis* in the study area, it is important to emphasize that the capture of this species is not common in Timóteo [7], which draws attention to the beginning of a possible adaptation and colonization of *Lutzomyia longipalpis* in the urban area of the municipality, as demonstrated in Northeast Brazil by Agra and Collaborators [21].

### 4.2. Detection of Leishmania spp. DNA

*Psychodopygus davisi* and *Pressatia choti* were the only ones detected with *Leishmania* spp. DNA in PERD. Special attention should be given as they deal with species that have already been reported with *Leishmania* infection. In the case of *Psychodopygus davisi*, there are records of infection by *Leishmania braziliensis* in the Amazon region, with reports that this species may be a likely vector in the region [22,23]. 

The report of *Pressatia choti* with detection of *Leishmania braziliensis* DNA is important, and has already been observed in a study carried out in northeastern Brazil [24]. This species is not considered a vector in the *Leishmania* transmission cycle, but is one of the most reported species in PERD [5]. 

It is important to highlight that there were records of TL cases of professionals and visitors after incursions on the trails of the PERD (information obtained through reports from PERD employees). These findings raise the hypothesis that essas especies may participate in the transmission cycle of *Leishmania braziliensis* in the study area. However, more detailed studies are needed to evaluate the potential of *Pressatia choti* e and *Psychodopygus davisi* as a vector.

Among the species with the presence of *Leishmania* spp. DNA in Timóteo, detection rates were higher than those reported in other studies. Oliveira-Pereira and collaborators [25], in the state of Maranhão, found a rate of 0.80% in females of *Nyssomyia whitmani*. Neitzke-Abreu and collaborators [26] worked in endemic regions of TL in the state of Paraná, and reported a detection rate of 1.12% in *Nyssomyia whitmani*.

Despite the large number of females of *Nyssomyia whitmani*, the highest rate of *Leishmania* spp. DNA detection was observed in *Migonemyia migonei*, a species already recognized as a vector, and which may be actively participating in the transmission cycle of *Leishmania* in the municipality together with *Nyssomyia whitmani* and *Nyssomyia intermedia*. It is possible, therefore, to suggest that these three species are also responsible for the transmission of *Leishmania* in the region.

Regarding the species *Evandromyia lenti* (DNA detection rate of 14.29%), *Evandromyia sallesi* (DNA detection rate of 3.13%), and *Micropygomyia quinquefer* (DNA detection rate of 0.88%), all had only a positive sample each, but as they presented a low number of captured specimens, the result of the DNA detection rate was high. This do not indicate that these species are responsible for the transmission of *Leishmania* in the area. However, we should not ignore these findings, even though these species are not reported as vectors.

*Micropygomyia quinquefer* has already been registered with *Leishmania braziliensis* DNA in a study carried out in Mato Grasso do Sul [27]. However, this information should be viewed with caution, as this species belongs to a group of sand flies in which females feed on cold-blooded animals [28].

The detection of *Leishmania* spp. DNA in *Evandromyia lenti* and *Evandromyia sallesi* has already been reported in other studies [27,29]. *Evandromyia sallesi* has already been reported to be naturally infected by *Leishmania braziliensis* in a study carried out in caves in the state of Minas Gerais [30]. 

The result obtained from *Nyssomyia intermedia*, found in Timóteo with *Leishmania guyanensis* DNA, may cause surprise, since *Leishmania guyanensis* is a species with a geographical distribution linked to the northern region of Brazil [31] and having *Nyssomyia umbratilis* as one of the main vectors [32]. However, there are studies reporting the presence of *Leishmania guyanensis* in other regions of Brazil, including the state of Minas Gerais [33,34]. The present study, in addition to being another record of the presence of *Leishmania guyanensis* in Minas Gerais, points to the possibility of *Nyssomyia intermedia* acting as a vector. However, more studies are needed to better demonstrate the role of *Leishmania guyanensis* in the study region.

This is the first report of de *Migonemyia migonei* with *Leishmania amazonensis* DNA in Brazil. Experimental infection data have shown that infection by *Leishmania amazonensis* in *Migonemyia migonei* is possible [35,36]. It is important to mention that *Leishmania amazonensis* have been recorded in Minas Gerais in sand flies and dogs [29,37]. Therefore, the present study suggests that *Leishmania amazonensis* may also be a possible agent of leishmaniasis in Timóteo.

Our results show that in the municipality of Timóteo there are at least three species of *Leishmania* with the possibility of maintenance of the life cycle, being able to have direct participation in cases of leishmaniasis both in the urban area and in the forested area (PERD) of the city, but with the possibility of different vectors between the two areas.

### 4.3. Blood Source Study

Among the samples analyzed and collected in PERD, two females of *Nyssomyia intermedia* with the presence of DNA of *Homo sapiens,* are samples that were collected on the edges of the forest. It is interesting to note that the edge of the forest is an area close to human habitation and the presence of human beings in this region is intense, which justifies the presence of female de *Nyssomyia intermedia* feeding on *Homo sapiens* blood. 

The other three female sand flies from PERD were collected inside the forest (approximately 1000 m away from the edge). The presence of DNA of *Dasyprocta leporina* (Agouti) was observed in a female of *Pressatia choti*, while DNA of *Dasypus novemcinctus* (Armadillo) and *Sphiggurus villosus* (Hedgehog) were observed in females of *Psychodopygus davisi*. The presence of DNA from these mammals in female sand flies, in addition to studies which reported the presence of natural infection by *Leishmania* in *Dasypus novemcinctus* [38] and *Sphiggurus villosus* [39], makes the hypothesis that these mammals are possible reservoirs of *Leishmania*. However, more studies are needed to confirm this hypothesis. 

In the present work, studies indicate a lower proportion of *Homo sapiens* compared to *Gallus gallus* (Chicken) (z = 4.276 *p* < 0.0001) and *Sus scrofa* (Pig) (z = 1.715 *p* < 0.05). The presence of a blood source in *Homo sapiens* in the urban area should be seen as normal, since the fed female sand flies were collected in the peri-domicile. These data are in agreement with others studies [40,41] who observed that sand flies from urban areas feed to a greater extent on chickens, humans, and also dogs. 

The role of birds in the epidemiology of leishmaniasis has been widely discussed, even though they are considered refractory to *Leishmania* infection. Tanure and collabora-tors [42] in the municipality of Governador Valadares verified that 43.6% of *Lutzomyia longipalpis* fed in *Gallus gallus*. *Sus scrofa*, as a mammal, draws attention to a possible participation in the *Leishmania* transmission cycle, given that there are records of infection by *Leishmania* in the state of Maranhão [43], in addition to the studies by Baum and collaborators [44], where using molecular analysis in females of *Nyssomyia intermedia* collected in the state of Paraná verified the presence of samples with DNA of *Sus scrofa*. However, it is important to highlight that more studies are needed to clarify the real role of *Sus scrofa* in the *Leishmania* cycle. 

The role of *Gallus gallus* and *Sus scrofa*, even though they are not *Leishmania* reservoirs, are important, since these species are a food source which help in maintaining the life cycle of sand flies in urban areas. The results presented here are essential to help the adoption of more effective measures to prevent and control leishmaniasis.

## 5. Conclusions

With this study, it was possible to identify the presence of three species of *Leishmania* in the study areas, *Leishmania braziliensis*, *Leishmania amazonensis*, and *Leishmania guyanensis*, with the possibility of *Leishmania braziliensis* being the main species responsible for the cases of tegumentary leishamaniasis in the region, since this species was identified in the most diverse sand fly samples collected.

The presence of *Leishmania braziliensis* DNA in *Pressatia choti* and *Psychodopygus davisi* in the PERD suggests a possible participation of these species in the *Leishmania* transmission cycle in the PERD. 

The large number of *Nyssomyia whitmani* in the urban area of Timóteo, associated with its presence of *Leishmania braziliensis* DNA, make this species the main vector in the municipality. 

These results may help the Health Authorities to adopt more effective measures to prevent and control Leishmaniasis in the municipality, such as, for example, intensifying the preventive campaigns no PERD and nas residencias do entorno do PERD. The results obtained from the study of blood sources, with the participation of *Gallus gallus* and *Sus scrofa*, are important and can be essential to help also the adoption of more effective measures for the prevention and control of leishmaniasis.

## Figures and Tables

**Table 1:** Numbers of sand flies according to species and sex captured in Timóteo, Minas Gerais state, Brazil, 2012–2014.

Species	Male	%^■^	Female	%^▲^	Total	%^●^
*Lutzomyia* sp.	0	0.00	1	0.04	1	0.03
*Brumptomyia* sp.	0	0.00	73	2.71	73	1.85
*Br. Avellari*	98	7.84	0	0.00	98	2.49
*Br. nitzulescui*	4	0.32	0	0.00	4	0.10
*Ev. cortelezzii*	1	0.08	0	0.00	1	0.03
*Ev. Lenti*	6	0.48	7	0.26	13	0.33
*Ev. Sallesi*	0	0.00	32	1.19	32	0.81
*Ev. termitophila*	0	0.00	4	0.15	4	0.10
*Lu. longipalpis*	4	0.32	3	0.11	7	0.18
*Mi. capixaba*	0	0.00	4	0.15	4	0.10
*Mi. quinquefer*	11	0.88	113	4.20	124	3.15
*Mg. migonei*	25	2.00	91	3.38	116	2.94
*Ny. whitmani*	837	66.96	1784	66.27	2621	66.49
*Ny. intermedia*	214	17.12	504	18.72	718	18.21
*Pi. bianchigalatiae*	0	0.00	1	0.04	1	0.03
*Pi. Fischeri*	21	1.68	23	0.85	44	1.12
*Pi. Pessoai*	15	1.20	4	0.15	19	0.48
*Pr. Choti*	9	0.72	31	1.15	40	1.01
*Pa. aragaoi*	0	0.00	2	0.07	2	0.05
*Pa. lutziana*	0	0.00	1	0.04	1	0.03
*Pa. pascalei*	2	0.16	1	0.04	3	0.08
*Ps. carreirai*	1	0.08	6	0.22	7	0.18
*Ps. Davisi*	1	0.08	6	0.22	7	0.18
*Ty. longispina*	1	0.08	1	0.04	2	0.05
Total (%) *	1250 (31.71%) *	100.00	2692 (68.29%) *	100.00	3942 (100.00%)	100.00

* Comparison between the proportions of captured sand flies according to sex: z = 32.48 *p* < 0.0001. %^■^ Frequency of male sand flies species collected. %^▲^ Frequency of female sand flies species collected. %^●^ Frequency of total sand flies species collected.

**Table 2:** DNA detection rate of *Leishmania* spp. in female sand flies according to species, collected in the PERD and in Timóteo, Minas Gerais state, Brazil, 2012–2014.

Species *	PERD					Timóteo					Total				
	N. of Females ^#^	%^■^	Samples Positive	%^▲^	DR	N. of Females ^#^	%^■^	Samples Positive	%^▲^	DR	N. of Females ^#^	%^■^	Samples Positive	%^▲^	DR
*Lutzomyia* sp. ●	1	0.27	0	-	-	1	0.04	1	-	-	2	0.07	1	1.75	-
*Ev. Lenti*	0	0.00	0	0.00	0.00	7	0.27	1	1.96	14.29	7	0.24	1	1.75	14.29
*Ev. Sallesi*	0	0.00	0	0.00	0.00	32	1.25	1	1.96	3.13	32	1.09	1	1.75	3.13
*Mg. migonei*	26	7.03	0	0.00	0.00	91	3.54	3	5.88	3.30	117	3.99	3	5.26	2.56
*Ny. whitmani*	23	6.22	0	0.00	0.00	1784	69.44	37	72.55	2.07	1807	61.61	37	64.91	2.05
*Ny. intermedia*	53	14.32	0	0.00	0.00	504	19.62	7	13.73	1.39	557	18.99	7	12.28	1.26
*Ps. Davisi*	154	41.62	2	33.33	1.30	6	0.23	0	0.00	0.00	160	5.46	2	3.51	1.25
*Pr. Choti*	105	28.38	4	66.67	3.81	31	1.21	0	0.00	0.00	136	4.64	4	7.02	2.94
*Mi. quinquefer*	8	2.16	0	0.00	0.00	113	4.40	1	1.96	0.88	121	4.13	1	1.75	0.83
Total	370	100.00	6	100.00	1.62	2569	100.00	51	100.00	1.94	2939	100.20	57	100.00	1.94

* Female sand fly species positive according to PCR/ITS1. DR—DNA Detection Rate. ^#^ Only female sand flies used in the molecular study for the detection of *Leishmania* spp. DNA. ● The DNA detection rate was not calculated, as it was not possible to define the species. %^■^ Frequency of female sand flies analyzed. %^▲^ Frequency of positive samples.

**Table 3:** Identification of *Leishmania* spp. DNA present in female sand flies collected in the PERD and in Timóteo, Minas Gerais state, Brazil, 2012–2014.

Leishmania Species	Sand Flies	Number and Frequency of Samples Collected					
		Timóteo	%^■^	PERD	%^▲^	Total	%^●^
*Leishmania (Viannia) guyanensis*	*Ny. Intermedia*	1	2.13	0	0.00	1	2.04
*Leishmania (Leishmania) amazonensis*	*Mg. migonei*	1	2.13	0	0.00	1	2.04
*Leishmania (Viannia) braziliensis*	*Mi. quinquefer*	1	2.13	0	0.00	1	2.04
*Leishmania (Viannia) braziliensis*	*Lutzomyia* sp.	1	2.13	0	0.00	1	2.04
*Leishmania (Viannia) braziliensis*	*Pr. Choti*	0	0.00	2	100.00	2	4.08
*Leishmania (Viannia) braziliensis*	*Ev. Sallesi*	1	2.13	0	0.00	1	2.04
*Leishmania (Viannia) braziliensis*	*Mg. migonei*	1	2.13	0	0.00	1	2.04
*Leishmania (Viannia) braziliensis*	*Ny. Whitmani*	35	74.47	0	0.00	35	71.43
*Leishmania (Viannia) braziliensis*	*Ny. Intermedia*	6	12.77	0	0.00	6	12.24
Total		47	100.00	2	100.00	49	100.00

%^■^ Frequency of samples collected in Timóteo. %^▲^ Frequency of samples collected in the PERD. %^●^ Frequency of total samples collected.

**Table 4:** Blood source identification in naturally fed female sand flies collected in the PERD and in Timóteo, Minas Gerais state, Brazil, 2012–2014.

Sand Flies	Blood Source (Species)	Number of Samples Collected			
		Timóteo	PERD	Total	%
*Pressatia choti*	*Dasyprocta leporina*	-	1	1	2.27
*Psychodopygus davisi*	*Dasypus novemcinctus*	-	1	1	2.27
*Psychodopygus davisi*	*Sphiggurus villosus*	-	1	1	2.27
*Pintomyia fischeri*	*Gallus gallus*	1	-	1	2.27
*Nyssomyia intermedia*	*Homo sapiens*	2	2	4	9.09
*Nyssomyia intermedia*	*Gallus gallus*	4	-	4	9.09
*Nyssomyia intermedia*	*Sus scrofa*	2	-	2	4.55
*Migonemyia migonei*	*Gallus gallus*	7	-	7	15.91
*Nyssomyia whitmani*	*Homo sapiens*	4	-	4	9.09
*Nyssomyia whitmani*	*Gallus gallus*	10	-	10	22.73
*Nyssomyia whitmani*	*Sus scrofa*	9	-	9	20.45
Total		39	5	44	100.0

% Frequency of total samples collected.

## Data Availability

All data generated or analyzed during this study are included in this published article. R.P.B. declare that the results/data/figures in this manuscript have not been published, nor are they under consideration for publication elsewhere.

## Abbreviations

TL: Tegumentary Leishmaniasis; VL: Visceral Leishmaniasis; WHO: World Health Organization; IBGE: Instituto Brasileiro de Geografia e Estatística; PERD: Parque Estadual do Rio Doce; DNA: Deoxyribonucleic Acid; ITS1: Internal Transcribed Spacer 1; PCR: Polymerase Chain Reaction; CytB: Cytochrome B; DMSO: Dimetilsulfóxido; CAPES: Coordenação de Aperfeiçoamento de Pessoal de Nível Superior; CNPq: Conselho Nacional de Desenvolvimento Científico e Tecnológico; SISBIO: Sistema de Autorização e Informação em Biodiversidade; IEF/MG: Instituto Estadual de Florestas de Minas Gerais; Br.: *Brumptomyia*; Ev.: *Evandromyia*; Lu.: *Lutzomyia*; Mi.: *Micropygomyia*; Mg.: *Migonemyia*; Ny.: *Nyssomyia*; Pi.: *Pintomyia*; Pa.: *Psathyromyia*; Pr.: *Pressatia*; Ps.: *Psychodopygus*; Sc.: *Sciopemyia*; Ty.: *Trichopygomyia*.
